# Variability of coherent and incoherent features of internal tides in the north South China Sea

**DOI:** 10.1038/s41598-020-68359-7

**Published:** 2020-07-31

**Authors:** Bingtian Li, Zexun Wei, Xinyi Wang, You Fu, Qingjun Fu, Juan Li, Xianqing Lv

**Affiliations:** 1 College of Ocean Science and Engineering, Shandong University of Science and Technology, Qingdao, China; 2 Key Laboratory of Marine Science and Numerical Modeling, First Institute of Oceanography, Ministry of Natural Resources of China, Qingdao, China; 3 Qingdao National Laboratory for Marine Science and Technology, Qingdao, China; 4 College of Computer Science and Engineering, Shandong University of Science and Technology, Qingdao, China; 5 Key Laboratory of Physical Oceanography, Qingdao Collaborative Innovation Center of Marine Science and Technology, Ocean University of China, Qingdao, China

**Keywords:** Physical oceanography, Ocean sciences

## Abstract

The coherent and incoherent features of internal tides (ITs) in the north South China Sea (SCS) are investigated based on observations and numerical simulations. The 11-month (from May 2011 to March 2012) moored current observations indicate that coherent semidiurnal ITs are obviously amplified, which can be attributed to the interference of ITs. Interference enhances coherent motions of semidiurnal ITs, but weakens those of diurnal ITs. Moreover, observations also show that semidiurnal ITs are more incoherent than diurnal ITs. Variations of vertical stratification and surface tide forcing can hardly affect the incoherence of ITs. The increase of incoherent signal is largely due to the influence of mesoscale eddies. Mesoscale eddies affect both amplitude and phase of ITs, making them more incoherent. Mesoscale eddies not only increase the intensity of background currents, but also induce horizontal variations of density. Variations of horizontal density and the influence of background currents lead to the increase of incoherent signals. And semidiural ITs are more sensitive to the influence of mesoscale eddies, making them more incoherent than diurnal counterparts. Incoherent ITs, which induce strong current shear, play essential roles in cascading tidal energy to small-scale motions, and contribute to turbulent mixing eventually. The findings help to better understand ITs and may offer reference for the improvement of parameterization of ocean turbulent mixing in the northern SCS.

## Introduction

Internal tides (ITs) are commonly generated in stratified oceans by barotropic tidal currents flowing over seamounts, ridges and continental shelf breaks^1–4^. As an essential intermediate step of tide-to-turbulence cascade, ITs play important roles in dissipating surface tidal energy and enhancing mixing, which contribute to deep-water circulation^5–10^. Munk found that the breaking of ITs provides nearly half of the energy necessary to maintain the global meridional overturning circulation^11^. The Luzon Strait (LS), which is featured by two north–south oriented ridges, connects the western Pacific and South China Sea (SCS). At the LS, strong diurnal and semidiurnal ITs are generated when barotropic tides flow over the double ridges^12^. ITs propagate westward into the SCS, making it a region with strong ITs^13–15^. Wang et al. found that the ITs radiating from the LS dominate the tidal dissipation in the SCS. Without the ITs generated at the LS, the dissipation in the SCS will be at least one order of magnitude smaller^16^.

Coherent ITs are phase-locked with barotropic tides at the generation site. Variability of coherent ITs is primarily explained by spring-neap cycles in barotropic tides. During their propagation, incoherence grows and ITs lose coherence to surface tides^3,4,17,18^ . In the Bay of Biscay, incoherent semidiurnal signals explain 30% of the total motions^19^. Eich et al. attributed the growing of incoherent semidiurnal ITs in the Mamala Bay to the influences of both variable stratification and mesoscale motions^17^. Many scales of motions, such as large-scale circulations, near-inertial waves and mesoscale eddies, are active in the SCS, which induce the complex background currents and stratification in the SCS and further modulate the incoherent feature of ITs^20–31^. Xu et al. analyzed moored current observations in the SCS and found that both diurnal and semidiurnal ITs contain stronger coherent signals than incoherent counterparts. They also suggested that semidiurnal ITs are more incoherent than diurnal ITs^3^. Liu et al. investigated ITs in the southern SCS and revealed that semidiurnal ITs are more incoherent than diurnal ITs^18^. Cao et al. examined the seasonal variations of coherent signals and found that coherent diurnal ITs are stronger in winter and summer than in spring and autumn, and stronger coherent semidiurnal ITs appear in spring and autumn^4^.

ITs produce disturbances to sea surface height (SSH). Therefore, ITs can be detected by the altimeter^32^. But altimeters can only detect the coherent ITs^33^. In other words, coherent features are essential in estimating ITs from the SSH. While incoherent ITs play important roles in cascading tidal energy to turbulent mixing. Cao et al. found incoherent ITs induced strong current shear in the SCS^34^. Liu et al. found incoherent ITs had high-mode structure^18^. High-mode ITs have large current shear, which eventually break and drive turbulent mixing^35^. Coherent ITs propagated thousands of kilometers without significant loss of energy. Coherent ITs appear to have weak dissipation, therefore they can hardly participant in ocean mixing^36^. Incoherent ITs make contributions to tide-induced mixing^37^. Investigating coherent and incoherent features of ITs in the northern SCS can not only provide better understanding of ITs, but also make contributions to the improvement of parameterization of ocean turbulent mixing in the northern SCS. In this study, 11-month (from May 2011 to March 2012) moored current observations are used to investigate variability of coherent and incoherent ITs in the north SCS. Then simulations based on the Massachusetts Institute of Technology General Circulation Model (MITgcm) are performed to better understand the coherent and incoherent variations. The paper is organized as follows. Methods, including observations and numerical simulations are introduced in “Methods”. Description and investigation of coherent and incoherent features of ITs in the northern SCS are shown in “Results”. Finally, the summary and discussion are presented in “Summary and discussion”.

## Methods

### Mooring data

**Figure 1:**
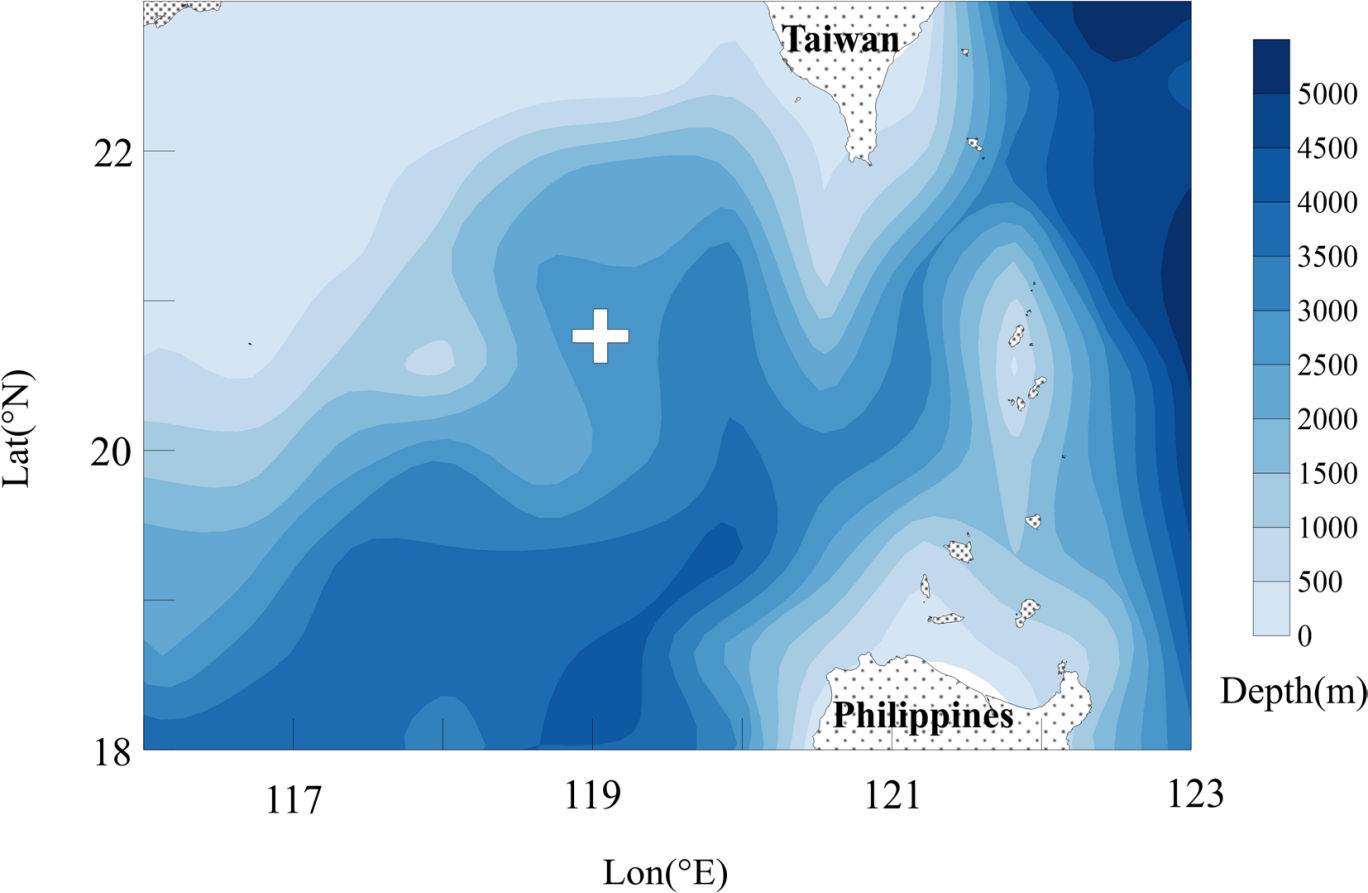
Topography of the northern SCS (the cross indicates mooring position).

As part of the SCS internal Wave Experiment, a sub-surface mooring (20.75°N, 119.00°E) which observed the ocean ranged from 70 to 450 m depths was deployed near the LS in the northern SCS^38^. Water depth of mooring place is 2,700 m. The velocity data were used for analysis. The velocity was measured by a 75 kHz upward-looking Acoustic Doppler Current Profiler (ADCP) with vertical resolution of 8 m and temporal interval of 3 min. The velocity data were interpolated onto uniform levels with 5 m intervals. The hourly averaged data from May 2011 to March 2012 were examined in this study. Mooring position and topography of the northern SCS are shown in Fig. 1.

### Observation data processing method

The barotropic currents can hardly be represented by depth-averaged currents because of the limitation of observation which do not cover the whole water depth. As a result, the barotropic tidal currents were extracted from the regional solution for the China Sea of the Oregon State University inverse barotropic tidal model (OTIS)^39^. The OTIS was proven to agree well with observations at the LS and in the northern SCS^40^. The baroclinic currents were calculated by removing the barotropic currents from the raw currents. Based on the least square method, harmonic analysis was applied to baroclinic currents to separate tidal components of diurnal (K_1_, O_1_, P_1_, Q_1_) and semidiurnal (M_2_, S_2_, N_2_, K_2_) ITs, which were coherent with surface tides at the LS. A fourth-order Butterworth filter was introduced to time series of baroclinic currents at diurnal (0.8–1.2 cpd) and semidiurnal (1.73–2.13 cpd) bands. Incoherent ITs were obtained by subtracting the coherent ITs from the band pass filtered baroclinic tidal currents. The kinetic energy (KE) of ITs for per unit volume, $KE = \frac{1}{2} \times \rho \times {(}u^{2} + v^{2} )$ (*ρ* = 1025 g/cm^3^ is the mean density of the sea water, *u* and *v* are filtered eastward and northward components of tidal currents) were calculated.

### Baroclinic tidal model configuration method

**Figure 2:**
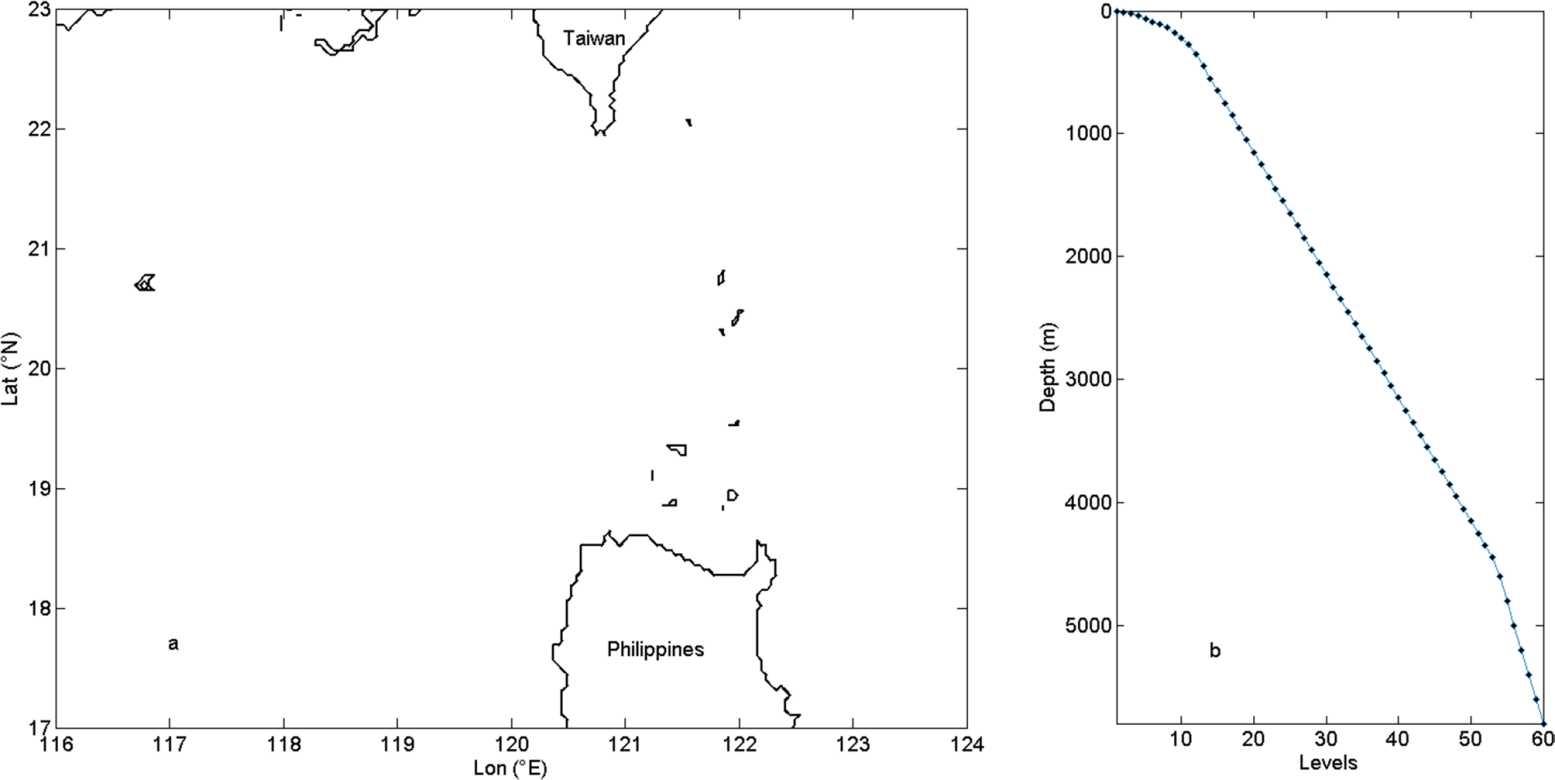
(**a**) The simulated domain and (**b**) depth of vertical levels.

MITgcm ocean model is used in this study to investigate the feature of ITs and the influence of mesoscale eddies on the incoherent signals. The model topography is from the General Bathymetric Chart of the Oceans (GEBCO_08) bathymetry data with a high resolution of 30 arcs. For internal tidal simulations, the simulation area includes the LS and part of the SCS (17°–23° N, 116°–124° E), which is shown in Fig. 2a and has a horizontal resolution of 1/24° × 1/24°. In the vertical direction (from 0 m at the top to 5,700 m at the bottom), there are 60 uneven vertical layers (Fig. 2b). The initial temperature and salinity profiles are derived from the monthly mean climatology of Generalized Digital Environmental Model, version 3 (GDEMv3). The initial fields are set to be horizontally homogeneous using temperature and salinity at site 20.50° N, 120.25° E. The model is forced by barotropic tidal currents at the open boundaries. Diurnal and semidiurnal ITs are simulated separately. For the diurnal ITs, only the dominant K_1_ and O_1_ are considered. For the semidiurnal ITs, the M_2_ and S_2_ are taken into consideration. The amplitudes and phases of these constituents are extracted from OTIS. A 0.5° width sponge layer is applied. For eddy-tide simulations which simulate both a mesoscale eddy and ITs, the initial fields are described according to Zhang et al.^41^. Details of eddy-tide experiments are shown in “Variation of incoherency”.

### Baroclinic tidal energy budget analysis method

The internal tidal energy is calculated according to Wang et al.^16^

1 $$TEN = - FD + C + ADV + DIS$$

where *TEN* represents the period-averaged tendency, FD is the period-averaged divergence of depth-integrated baroclinic energy flux, *C* is period-averaged and depth-integrated conversion of barotropic to baroclinic energy, *ADV* is the item of period-averaged advection and DIS is the period-averaged dissipation of baroclinic energy. When the simulation is stable, the simulated energy in the model is no longer varying, and TEN and ADV can be negligible. The equation can be approximated as

2 $$DIS = - C + FD,$$

*C* and *FD* are given as

3 $$C = g\int_{ - H}^{\varsigma } {\rho^{\prime}wbtdz} ,$$

4 $$FD = \nabla_{h} \cdot \left( {\int_{ - H}^{\varsigma } {{\mathbf{u}}^{\prime } p^{\prime } dz} } \right),$$

where *g* is the acceleration of gravity, *ξ* is the time-mean sea level, *H* is the water depth, $\rho^{\prime}$ is the density perturbation, $wbt$ is the vertical velocity of barotropic tides, ${\mathbf{u^{\prime}}}$ and $p^{\prime}$ are the horizontal baroclinic velocity ($u$ and $v$ for the eastward and northward direction respectively) and pressure perturbation. $\rho^{\prime}$ and $wbt$ can be calculated as

5 $$\rho^{\prime}(z,t) = \rho (z,t) - \overline{\rho }(z),$$

6 $$wbt = u\left( {\sigma \frac{\partial D}{{\partial x}}} \right. + \left. {\frac{\partial \varsigma }{{\partial x}}} \right) + v\left( {\sigma \frac{\partial D}{{\partial y}}} \right. + \left. {\frac{\partial \varsigma }{{\partial y}}} \right) + (\sigma + 1)\frac{\partial \varsigma }{{\partial t}},$$

where $\rho$ is the instantaneous density, $\overline{\rho }$ is the density averaged in a tidal period, $D = H + \zeta$ is the total water depth, and $\sigma$ is defined as $\sigma = (z - \zeta )/D,$*.* ${\mathbf{u^{\prime}}}$ and $p^{\prime}$ are given as

7 $$p^{\prime}(z,t) = - \frac{1}{H}\int_{ - H}^{\varsigma } {\int_{z}^{\varsigma } {g\rho^{\prime}(\overset{\lower0.5em\hbox{$\smash{\scriptscriptstyle\frown}$}}{z} ,t)} } d\overset{\lower0.5em\hbox{$\smash{\scriptscriptstyle\frown}$}}{z} dz + \int_{z}^{\varsigma } {g\rho^{\prime}(\overset{\lower0.5em\hbox{$\smash{\scriptscriptstyle\frown}$}}{z} ,t)} d\overset{\lower0.5em\hbox{$\smash{\scriptscriptstyle\frown}$}}{z} ,$$

8 $${\mathbf{u^{\prime}}}(z,t) = u(z,t) - \overline{u}(z) - \frac{1}{H}\int_{{{ - }H}}^{\varsigma } {\left[ {u(z,t) - \overline{u}(z)} \right]} dz.$$

where $\overline{u}$ is the velocity averaged in a tidal period.

## Results

### Basic properties

**Figure 3:**
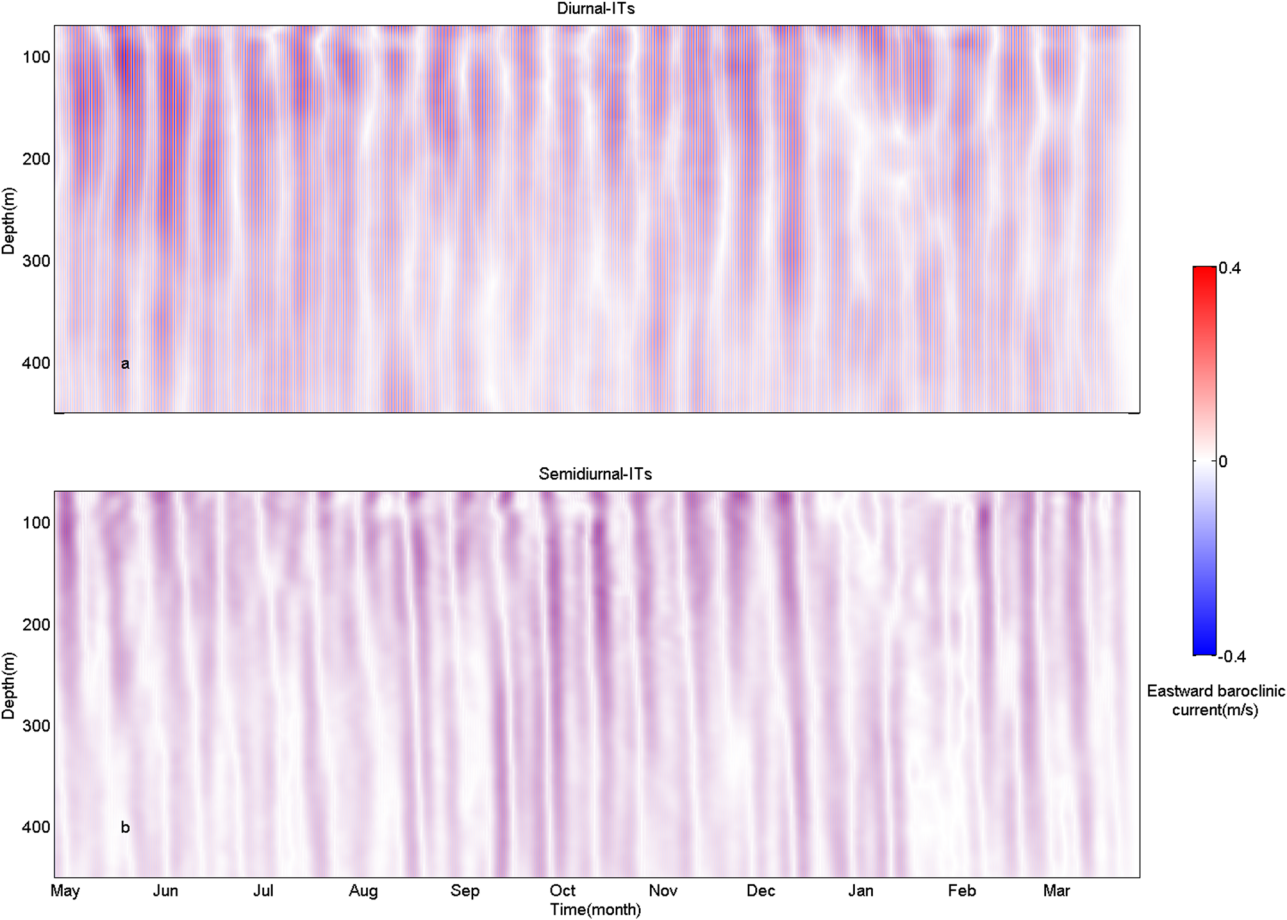
Distributions of baroclinic current velocities at (**a**) diurnal and (**b**) semidiurnal frequency bands at the mooring site.

Distributions of baroclinic current velocities at diurnal and semidiurnal frequency bands are displayed in Fig. 3a,b, respectively. In the northern SCS, diurnal ITs are stronger than semidiurnal ITs. Diurnal ITs follow obvious spring-neap cycles, which has strong 14-day periodic variations. While semidiurnal ITs nearly lose that feature in most of the observation period. In vertical, within the ADCP observing depths, diurnal baroclinic tidal currents tend to be obviously surface-intensified with larger velocities appearing above 250 m depth. But surface-intensification of semidiurnal ITs is not as obvious as that of diurnal ITs at the mooring site.

**Figure 4:**
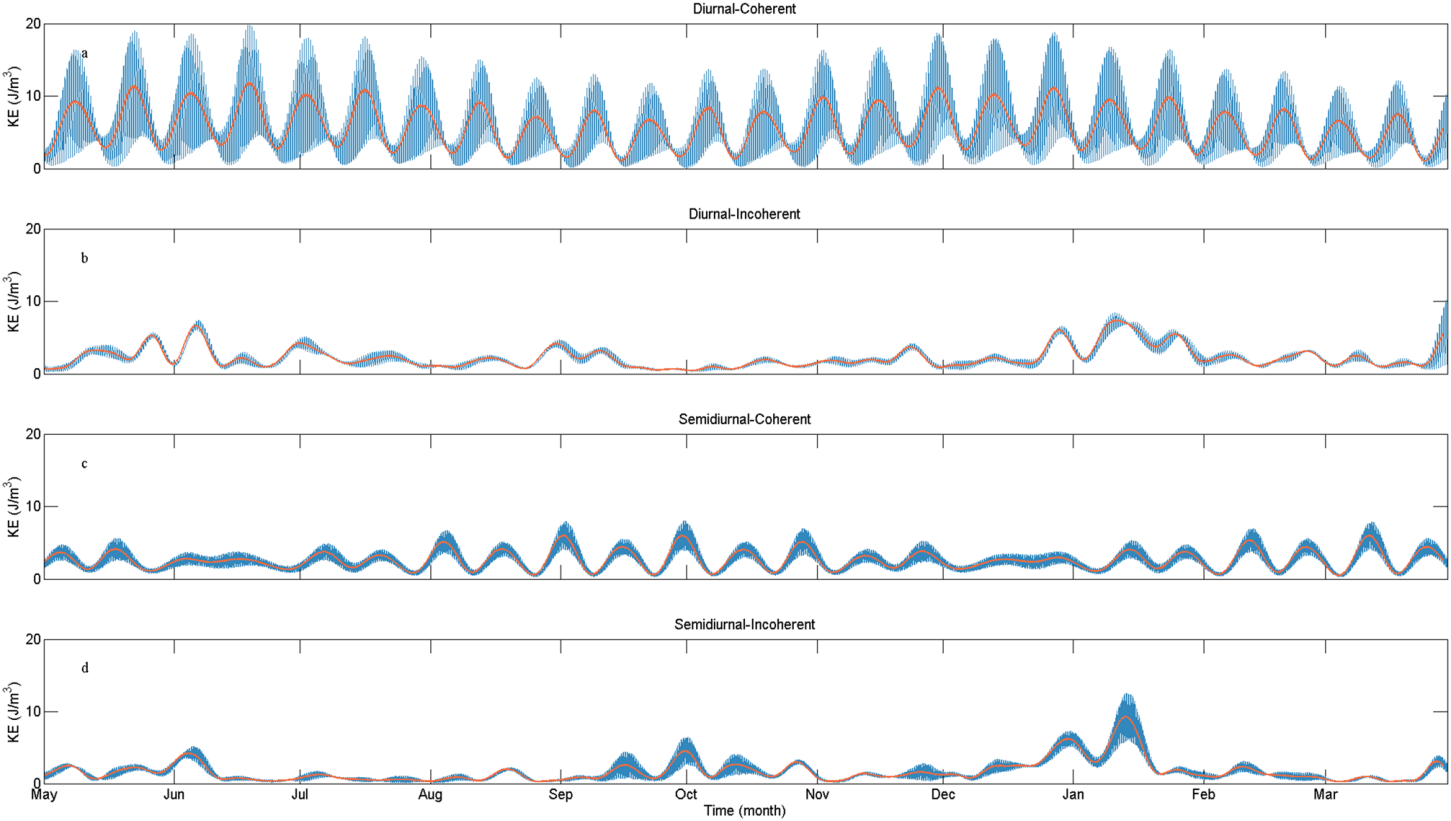
Time series of vertically averaged coherent and incoherent baroclinicKE (blue lines, orange lines present period-smoothed KE) at diurnal and semidiurnal frequency bands at the mooring stie.

**Figure 5:**
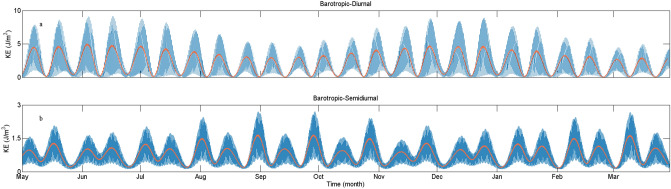
Time series of vertically averaged diurnal and semidiurnal barotropic KE (blue lines, orange lines present period-smoothed KE) at the LS (20.75° N, 121.50° E).

Vertically averaged KE of coherent and incoherent signals for diurnal and semidiurnal ITs are shown in Fig. 4. The coherent diurnal ITs are composed of K_1_, O_1_, P_1_, Q_1_, and the coherent semidiurnal considered M_2_, S_2_, N_2_, K_2_. The KE of corresponding barotropic tidal currents at the LS (20.75° N, 121.50° E) are shown in Fig. 5. The results indicate that variability of both coherent diurnal and semidiurnal ITs can be largely explained by the barotropic tidal forcing at the LS. Incoherent signals of both diurnal and semidiurnal ITs are not phase-locked to the surface tides and exhibit intermittent behaviors. Coherent diurnal ITs account for nearly 74% of diurnal KE, while the semidiurnal tidal currents contain only about 61% of coherent signals. In other words, incoherent signals can explain 26% and 39% of diurnal and semidiurnal KE, respectively. In the northern SCS, semidiurnal ITs are more incoherent than diurnal ITs.

For diurnal ITs, time-averaged KE of barotropic tide at the LS is 2.0 J/m^3^, and the corresponding KE of the coherent baroclinic signals at the mooring site is 5.6 J/m^3^. For semidiurnal ITs, time-averaged KE for barotropic tide at the LS is only 0.7 J/m^3^. KE of coherent semidiurnal ITs raises to 2.6 J/m^3^. Ratios of coherent baroclinic KE to barotropic KE are 2.8 and 3.7 for diurnal and semidiurnal ITs, respectively. It is interesting to note that the strength of coherent semidiurnal ITs at the mooring site is obviously amplified.

### Variation of coherency

It is notable that the strength of coherent semidiurnal ITs in the northern SCS is amplified. Observations show that coherent diurnal ITs account for 74% of diurnal KE, and the semidiurnal tidal currents contain 61% of coherent signals. More than 60% of both diurnal and semidiurnal ITs can be explained by coherent signals. In other words, variations of baroclinic tides will obviously cause the corresponding changes of coherent signals. Therefore, the increase of coherent intensity can be explained by the amplification of baroclinic tides. The internal wave regime is classified by the parameter of criticality (CR). CR is a nondimensional parameter, which represents the possibility of generating of ITs. ITs are more likely to generate in regions with CR larger than 1

9 $$CR = \left| { - \frac{\partial H(x)}{{\partial x}}\left( {\frac{{\omega_{tide}^{2} - f^{2} }}{{N^{2} (z) - \omega_{tide}^{2} }}} \right)^{{ - \frac{1}{2}}} } \right|$$

**Figure 6:**
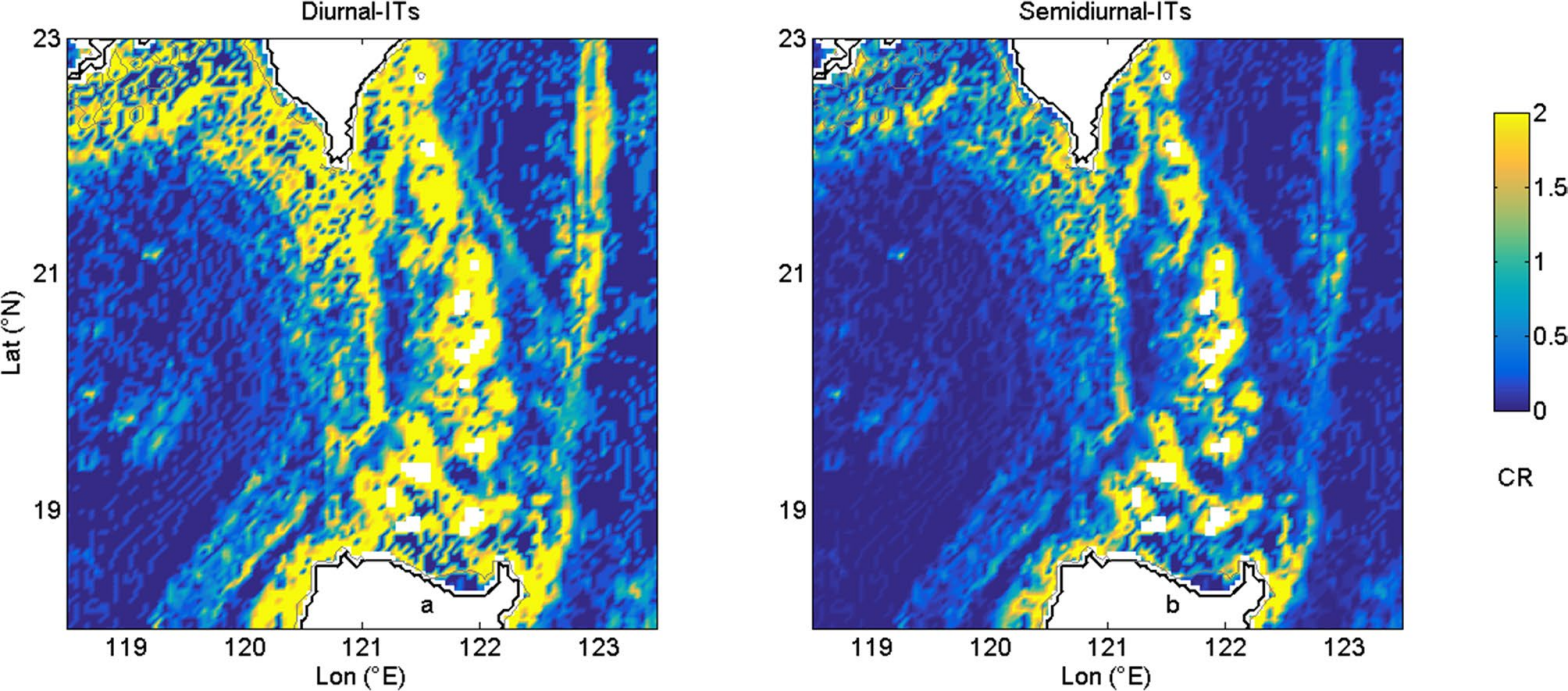
Criticality (CR) at the sea bottom of the LS for (**a**) diurnal and (**b**) semidiurnal ITs.

*H* is the water depth, $\omega_{tide}$ is frequencies for ITs, *f* is the Coriolis frequency and *N* is the buoyancy frequency.CR at the sea bottom of the LS for diurnal and semidiurnal ITs are shown in Fig. 6. CR of diurnal ITs is larger than that of semidiurnal ITs. The topography is more favor of generating ITs in the diurnal frequency bands than those in the semidiurnal frequency bands, which is incapable of explaining the amplification of semidiurnal ITs. Therefore, to better understand those variations, numerical modellings based on MITgcm are used to simulate the diurnal and semidiurnal ITs respectively. Simulations are conducted with stratification of January and July, which are regard as winter and summer runs respectively.

**Figure 7:**
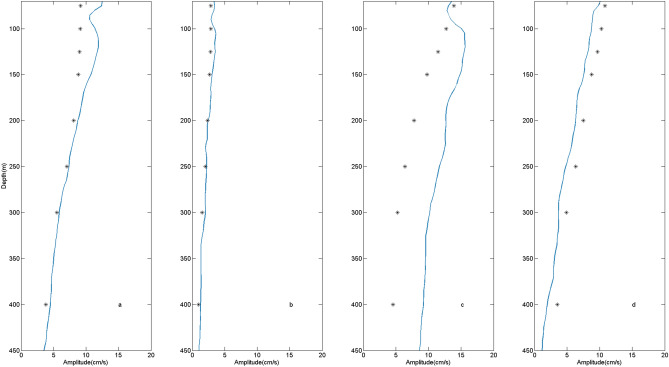
Winter and summer runs averaged eastward baroclinic tidal amplitudes of simulations (black asterisk) as well as those of observations (blue line) at frequency of (**a**) M_2_ and (**b**) S_2_ (**c**) K_1_ and (**d**) O_1_ at the mooring site.

Figure 7 shows comparison of simulated amplitudes, which are averaged of winter and summer runs, and observed results of eastward velocities of M_2_, S_2_, K_1_ and O_1_ at the mooring site. Table 1 displays averaged differences (vertical mean absolute errors of amplitudes) between observations and simulations. For semidiurnal ITs, errors of S_2_ are smaller than those of M_2_. For diurnal ITs, the simulated amplitudes of K_1_ is weaker than observation and making the difference larger than other constituents. Whereas the simulated O_1_ is much closer to observation. The enhancement of K_1_ has been observed by previous investigations, which can be attributed to the intrusion of Kuroshio^12^. In our simulation, the Kuroshio are not involved in the model, which probably leads to the larger error of K_1_.

**Table 1:** Winter and summer runs averaged differences (vertical mean absolute errors) of M_2_, S_2_, K_1_ and O_1_ eastward tidal current amplitudes between observations and simulations.

Internal tide	M_2_	S_2_	K_1_	O_1_
Amplitude difference (cm/s)	1.5	0.4	4.0	1.3

According to our simulated results, during the period of spring-tide in winter,there are 17.8 GW and 14.1 GW of energy transferring from barotropic to baroclinic tides for diurnal and semidiurnal ITs at the LS. The energy dissipates for diurnal and semidiurnal ITs is − 5.1 GW and − 5.5 GW, respectively. In winter, 29% of baroclinic tidal energy of diurnal ITs and 39% of baroclinic tidal energy of semidiurnal ITs are locally dissipated at the LS. In summer, generation rates for diurnal and semidiurnal ITs are 18.8 GW and 14.5 GW, respectively. And dissipation rates are − 5.6 GW and − 5.9 GW. Proportions of energy dissipate in summer are 30% and 41% for diurnal and semidiurnal ITs, respectively. Energy generated are obviously larger than those dissipated at the LS, therefore intensity of baroclinic tides are dominated by the conversion. Our simulated baroclinic energy budget is similar to Alford et al. and Simmons et al., which also indicates the accuracy of simulations^40,42^.

**Figure 8:**
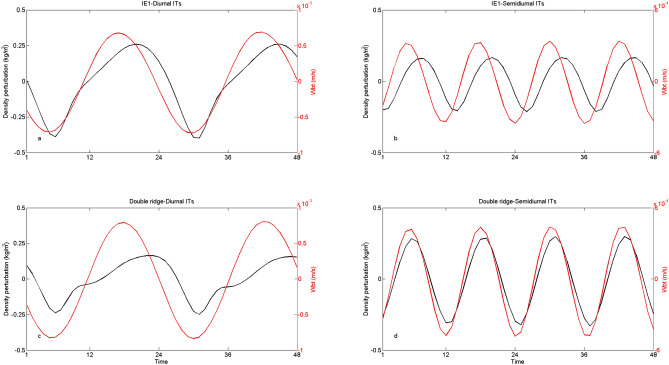
Density perturbation and vertical barotropic velocity at the depth of 125 m in IE1 of winter run for (**a**) diurnal and (**b**) semidiurnal ITs, as well as those (**c**, **d**) in the double-ridge simulation of winter run.

Idealized twin experiments are carried out to further investigate the difference between diurnal and semidiurnal ITs. Idealized experiment 1 (IE1) is designed with a single east ridge. In IE1, depth in region of the west ridge is set to be 3,600 m. Idealized experiment 2 (IE2) is carried out with a single west ridge. In IE 2, the east ridge is removed and the depth is set to be 3,700 m. In IE1 and IE2, model setups are the same as those in the double-ridge simulation except for the topography. Area-integrated conversion of baroclinic tidal energy are shown in Table 2. For both diurnal and semidiurnal ITs, conversions at the east ridge of the LS (IE1) are much larger than those at the west ridge (IE2), which indicates that ITs are largely generated on the east ridge. The distribution of CR also indicates that both diurnal and semidiurnal ITs are largely generated at the east ridge owing to the larger topography gradient (Fig. 6). It is notable that the conversion rates in the double-ridge simulation are much larger than the sums of single east and single west simulations for semidiurnal ITs in both winter and summer, which can be attributed to the resonance of semidiurnal ITs at the LS^43,44^. At the LS, tidal waves from the opposing ridges interfere with each other, influencing the conversion of ITs. The phase difference between the remote generated ITs and local baroclinic flow can either enhance or weaken the barotropic to baroclinic energy conversion compared to when remote generated ITs are absent^22^. Not only the semidiurnal ITs, but the diurnal ITs can also be affected by interference. However, interference of diurnal ITs at the LS is rarely discussed. Results show that in both winter and summer, conversions of diurnal internal tidal energy in the double-ridge cases are much smaller than the sums of single ridge cases, suggesting interference of diurnal ITs weakens the generation of diurnal ITs. Figure 8 shows density perturbation and vertical barotropic velocity at the depth of 125 m for both diurnal and semidiurnal ITs in the central of the LS (20.5°N, 121.7°E) during spring-tide period of winter (those in summer are similar,which are not shown). The conversion of ITs is largely governed by the amplitude of density perturbation and the phase difference between density perturbation and vertical barotropic velocity. For diurnal ITs, the interference decreases the conversion by weakening the amplitude of density perturbation and enlarging the phase difference. Whereas, the resonance of semidiurnal ITs enhances the conversion mainly by engendering the density perturbation more in phase with the local barotropic tide. Interference weakens the conversion of diurnal ITs but strengthen the generation of semidiurnal ITs, which leads to the amplification of coherent semidiurnal baroclinic tides.

**Table 2:** Area-integrated conversion (GW) of baroclinic tidal energy.

Season	ITs	Single east ridge (IE1)	Single west ridge (IE2)	East + west ridges	Double-ridge
Winter	Diurnal	18.0	5.4	23.4	17.8
	Semidiurnal	7.6	1.4	9.0	14.1
Summer	Diurnal	19.1	5.4	24.5	18.8
	Semidiurnal	7.8	1.5	9.3	14.5

### Variation of incoherency

Observations reveal that semidiurnal ITs contain 39% of incoherent signals, while diurnal tidal currents contain only 26% of incoherent signals. Semidiurnal ITs are more incoherent than diurnal counterparts within the observation period. Xu et al. and Cao et al. also found semidiurnal ITs were more incoherent than diurnal ITs^3,4^. However, those differences of incoherence are rarely investigated and the underlying mechanism remains unclear. Incoherence of ITs can be influenced by background currents and stratification during propagation. Incoherent ITs are extracted from simulations of both winter and summer runs: Harmonic analysis was applied to simulated currents to obtain coherent signals for ITs. Band-pass filter was introduced to time series at diurnal and semidiurnal bands. Incoherent ITs were obtained by subtracting the coherent ITs from the band pass filtered baroclinic tidal currents. Simulated results of last 10 days are used to extract the incoherent ITs. To avoid errors from band pass filter, results at the two ends (36 h for each end) are not considered for investigation, only results in the 7 middle days are used. Table 3 shows simulated incoherence of diurnal and semidiurnal ITs at the mooring site. Incoherent ITs remain weak and vary slightly from winter and summer runs, indicating that variation of vertical stratification or surface tide forcing has little effect on the incoherent signals of ITs.

**Table 3:** Vertically averaged simulated incoherence of diurnal and semidiurnal ITs in winter and summer runs at the mooring site.

Season	Winter		Summer	
ITs	Diurnal	Semidiurnal	Diurnal	Semidiurnal
Incoherence	4%	6%	2%	4%

**Figure 9:**
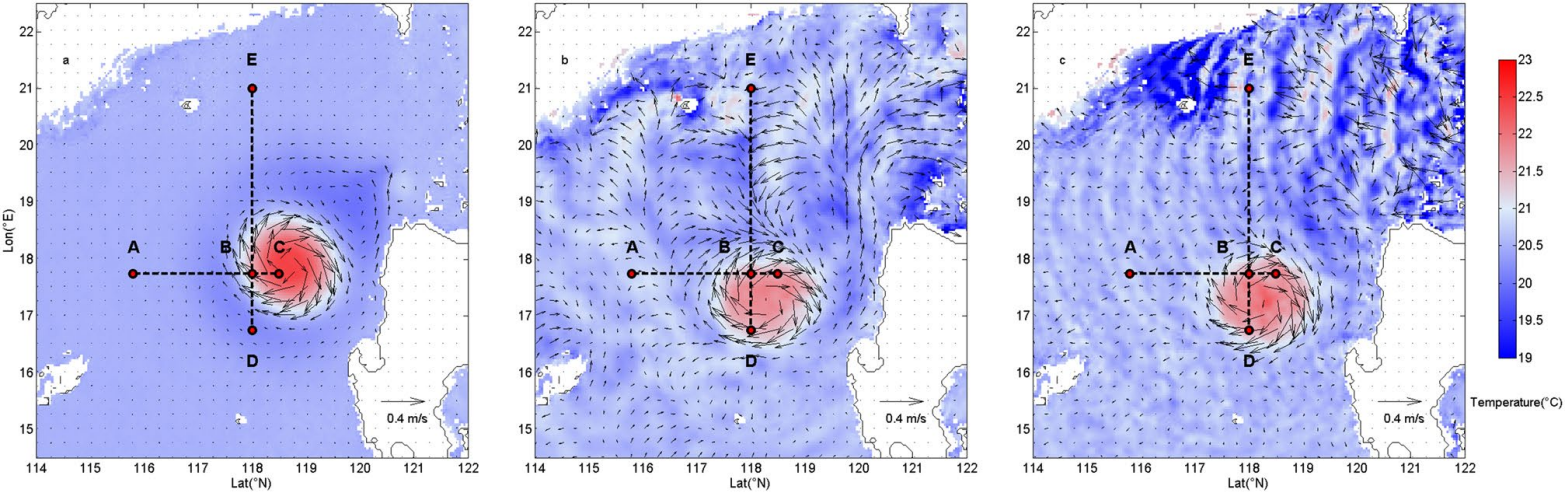
Distribution of temperature and velocity at 100 m depth at the last hour of (**a**) step1 and step 2 for (**b**) diurnal and (**b**) semidiurnal ITs (A–E indicate the eddy-free and eddy-influence locations).

Background currents, such as mesoscale eddies, can have an obvious impact on the incoherence of ITs. The SCS is abundant with strong mesoscale activities^30,38^, and anticyclonic eddies (AE) are more frequently observed than cyclonic eddies^45^. Idealized experiment 3 (IE3) is carried out to further investigate the influence of an idealized AE on the incoherence of ITs. The horizontal and vertical structure are described by the universal structures of mesoscale eddies according to Zhang et al.^41^. Mesoscale eddies which are reconstructed using the universal structures have been proven close to observation and been applied to investigate the oceanic mass transport by eddies^46^. In IE3 an AE with horizontal radius of 80 km, vertical scale of 500 m is centered at (18° N, 119° E). The IE 3, which runs for 35 days, is simulated in two steps. The first step is tide-free simulation, which only simulates AE. After running for 15 days, the simulation is stable. The temperature anomaly (in the center of AE) at 100 m depth is 1.9 °C and the maximum velocity of the AE is about 0.7 m/s (Fig. 9a). In the next 20 days of simulation, tides are added at the open boundary, then the model is simulated with both the AE and tides. Results of the last 10 days of step 2 are used for analysis. Since the eddy moves southwestward when tides are performed, the simulation area in IE 3 is larger (12°–24°N, 110°–125°E). Figure 9b,c show the temperature and velocity fields at end of step 2 for diurnal and semidiurnal ITs, respectively. Even tough ITs induce disturbance to the temperature field, the evident anomaly is caused by the AE. Both diurnal and semidiurnal ITs make the AE move southwestward.

Incoherence of eddy-influence (sites B–D in Fig. 9) and eddy-free locations (sites A and E in Fig. 9) are shown in Table 4. For sites A and E, incoherence of both diurnal and semidiurnal ITs are relatively weak which are similar to the winter and summer runs. While at eddy-influence locations (sites B–D) incoherence of ITs increase evidently, which suggests that mesoscale eddies can effectively cause the incoherence of both diurnal and semidiurnal ITs. For all the eddy-influence locations, semidiurnal ITs are more incoherent than diurnal ITs, suggesting that incoherent semidiurnal ITs are more sensitive to the eddy than diurnal counterparts.

**Table 4:** Vertically averaged incoherence of simulated diurnal and semidiurnal ITs in IE3 at eddy-influence and eddy-free locations.

location	A (%)	B (%)	C (%)	D (%)	E (%)
Incoherence of diurnal ITs	4	12	17	9	4
Incoherence of semidiurnal ITs	5	14	20	14	5

**Figure 10:**
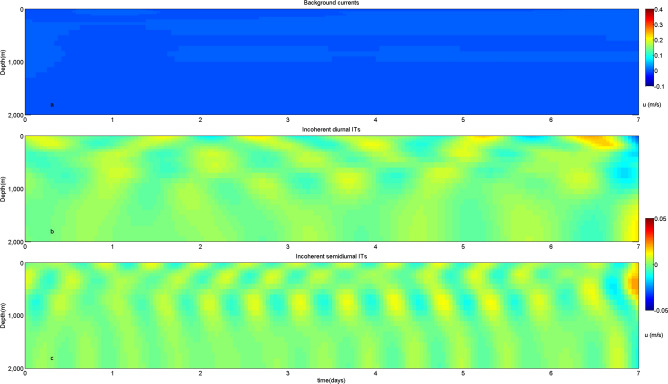
Distributions of eastward velocities of (**a**) background currents, (**b**) incoherent diurnal ITs and (**c**) incoherent semidiurnal ITs at site A.

**Figure 11:**
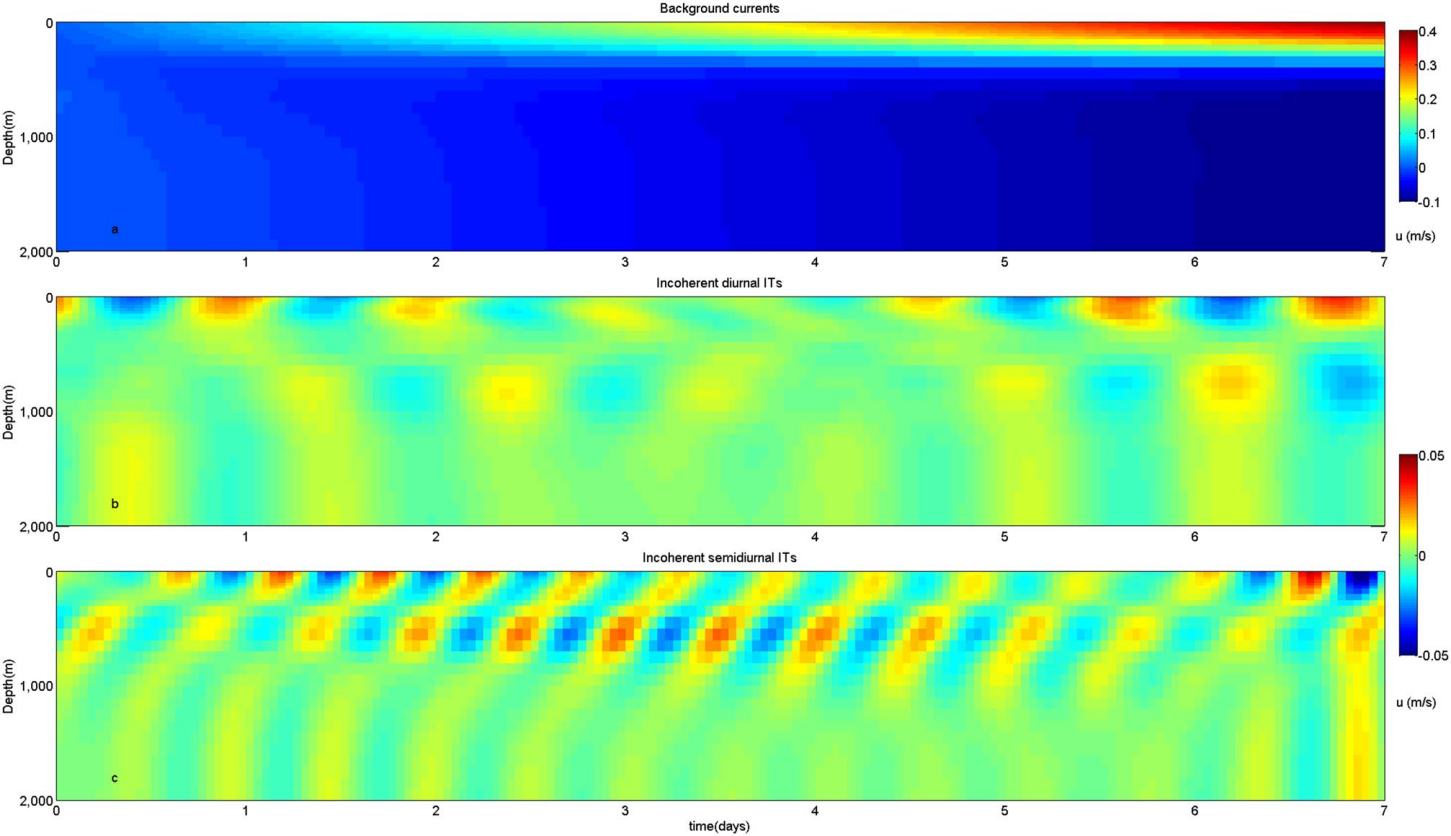
Distributions of eastward velocities of (**a**) background currents, (**b**) incoherent diurnal ITs and (**c**) incoherent semidiurnal ITs at site C.

Then, the simulated time series are low-pass filtered using a fourth-order Butterworth filter with a cutoff frequency of 0.4 cpd to obtain velocities of background currents, which are caused by the AE. Eastward velocities of background currents and incoherent ITs at site A are shown in Fig. 10 (results of the northward velocities are similar and not shown). Site A is remote from the AE, which is not influenced by the eddy, the background currents are extremely weak at this site. Both incoherent diurnal and semidiurnal ITs remain weak at this site. Figure 11 shows eastward velocities of background currents and incoherent ITs at site C (results of the northward velocities are similar and not shown). Mesoscale eddies can cause temperature and salinity anomalies and induce horizontal variations of density. In the beginning of simulation step 2, site C is near the center of the AE, which results in strong variation of horizontal density and weak background currents. Then the intensity of background currents increases with the AE moving southwestward. At the end of simulation, site C is near the edge of the AE, and the intensity of background currents becomes evident. Both diurnal and semidiurnal ITs exhibit obvious incoherent signals during the entire time, indicating that variation of horizontal density and influence of background currents lead to the incoherence of ITs.

**Figure 12:**
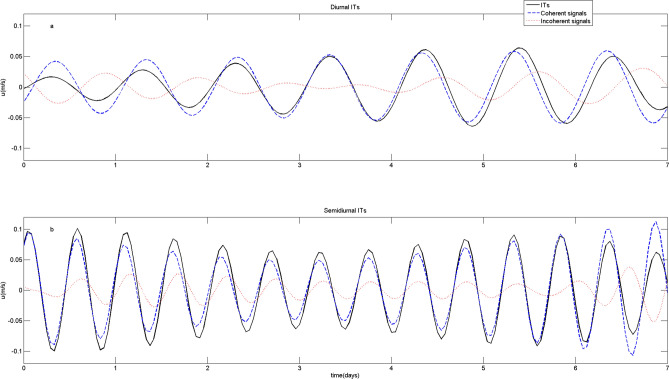
Eastward velocities of baroclinic tides (black solid line), coherent signals (blue dashed line) and incoherent signals (red dot line) at the depth of 50 m for (**a**) diurnal and (**b**) semidiurnal ITs at site C.

Figure 12 shows eastward velocities of ITs, coherent signals and incoherent signals at the depth of 50 m. The AE increases the incoherency by affecting both the amplitude and phase of ITs. We notice that the simulated incoherence is weaker than observation. The background currents of the real ocean are more complicate. This may be one of the reasons why the observed incoherence is stronger.

## Summary and discussion

Combining observations with numerical simulations, coherent and incoherent features of ITs in the north SCS are investigated. Observations of 11-month moored currents reveal that coherent semidiurnal ITs are obviously amplified compared to those of diurnal counterparts. Numerical simulations indicate interference of baroclinic tides weakens the conversion of baroclinic diurnal energy but enhances that of semidiurnal ITs. The conversion of diurnal ITs is undermined by both impairing the amplitude of density perturbation and increasing the phase difference between density perturbation and vertical barotropic velocity. Whereas resonance of semidiurnal ITs can effectively enhance the conversion of semidiurnal ITs by engendering the density perturbation more in phase with the barotropic tide. According to observations, more than 60% of the tidal motions can be explained by coherent signals for both diurnal and semidiurnal ITs. Variations of baroclinic tide intensity will obviously cause the corresponding changes of coherent signal. Therefore, interference of ITs which weakens the intensity of diurnal ITs but enhances that of semidiurnal ITs, contributes to the amplification of semidiurnal coherent signals.

Observations also show that semidiurnal ITs are more incoherent than diurnal counterparts. The simulated incoherence is weak if the initial fields are horizontally homogeneous. Variations of vertical stratification and surface tide forcing cannot obviously increase the incoherence of both diurnal and semidiurnal ITs. Mesoscale eddies can effectively cause strong incoherence by both influence the amplitude and phase of ITs. Because the appearance of mesoscale eddies is largely stochastic, then incoherent ITs exhibit intermittent behaviors. The AE not only increases the intensity of background currents, but also induces horizontal variations of density. Variations of horizontal density and the influence of background currents lead to the increase of incoherent ITs. And semidiural ITs are more sensitive to the influence of mesoscale eddies, making them more incoherent than the diurnal counterparts. Because only coherent ITs can be detected by altimeters and semidiurnal ITs are less coherent, then energy or energy fluxes of semidiurnal ITs estimated using SSH data may not as accurate as that of diurnal ITs in the northern SCS. Given that incoherent ITs contribute to the enhancement of turbulent mixing. Semidiurnal ITs can make more contribution to tide-induced mixing in the northern SCS. The existence of mesoscale eddies further cascades energy of ITs to small-scale motions, eventually to turbulent mixing.

## Data Availability

The data analyzed in this study can be obtained at this website (https://jumpshare.com/b/fbdLZwqao4OkUR8421l9).
